# How Computational Chemistry and Drug Delivery Techniques Can Support the Development of New Anticancer Drugs

**DOI:** 10.3390/molecules25071756

**Published:** 2020-04-10

**Authors:** Mariangela Garofalo, Giovanni Grazioso, Andrea Cavalli, Jacopo Sgrignani

**Affiliations:** 1 Department of Pharmaceutical and Pharmacological Sciences, University of Padova, 35131 Padova, Italy; 2 Department of Pharmaceutical Sciences, University of Milano, 20133 Milan, Italy; giovanni.grazioso@unimi.it; 3 Swiss Institute of Bioinformatics, 1015 Lausanne, Switzerland; andrea.cavalli@irb.usi.ch; 4 Institute for Research in Biomedicine (IRB), Università della Svizzera Italiana (USI), 6500 Bellinzona, Switzerland

**Keywords:** drug delivery, computational chemistry, anticancer drug design

## Abstract

The early and late development of new anticancer drugs, small molecules or peptides can be slowed down by some issues such as poor selectivity for the target or poor ADME properties. Computer-aided drug design (CADD) and target drug delivery (TDD) techniques, although apparently far from each other, are two research fields that can give a significant contribution to overcome these problems. Their combination may provide mechanistic understanding resulting in a synergy that makes possible the rational design of novel anticancer based therapies. Herein, we aim to discuss selected applications, some also from our research experience, in the fields of anticancer small organic drugs and peptides.

## 1. Introduction

Despite huge advances in the development of novel therapeutic approaches, cancer is one of the leading causes of death worldwide; every year millions of people are diagnosed with oncological pathologies [1] and more than half of them will die from it [2].

Small organic drugs are still the primary source of anticancer therapies counting the approval of six new chemical entities by the food and drug administration in 2019 [3].

At the same time, while the role of peptides in the development of innovative therapies and new diagnostic tools for oncological diseases is still marginal, it is important to note that in 2018 about 35 peptides were in different stages of clinical testing and four molecules have been approved for the clinical use from 2000 to 2016 [4].

Synthesis and the subsequent biological tests are the heart of the anticancer drug development process, however two other research fields, apparently far from each other, such as computational chemistry and drug delivery, can significantly contribute to the success of new therapeutic strategies. Computational methods, for example consolidated simulation techniques, such as molecular dynamics (MD), docking, free energy calculations, chemoinformatic and machine learning algorithms are mainly used to: i) design new chemical entities able to bind to a given target and ii) to improve the selectivity of known hit or lead compounds [5,6,7,8,9,10,11,12,13]. Combinations of the same computational approaches can be also employed to improve the ability of the molecules to penetrate the cells or to resist to metabolism [14,15,16,17,18,19].

Concerning targeted drug delivery (TDD), it represents a promising approach to improve treatment outcomes through the delivery of the administered drugs to the target tissues, while eliminating or minimizing its accumulation at any non-target sites. Nevertheless, achieving therapeutically relevant drug concentrations in the tumor mass, especially in the case of solid tumors, for a time sufficient to allow the therapeutic activity of the drug, is challenging. Furthermore, high dose therapy required to maintain the remission causes systemic side effects, forcing the discontinuation of therapy in many patients. Thus, targeting drugs with designed drug delivery systems offers the option to enhance the therapeutic efficacy and to reduce systemic toxicity of anti-cancer agents.

The combination of the two different research fields in this work, can probably surprise the reader, however, we do hope that this can inspire the design of innovative scientific projects focused on drug development and to the design of innovative therapies.

In the following, we will present and discuss some selected examples, including some from our research experience: (1) to make more evident the real impact of computational chemistry and drug delivery techniques in the anticancer therapies′ development and (2) to explain how these two disciplines can directly interact.

Overall, we wish that this review could help scientists from other research fields e.g., medicinal chemists or pharmacologists to interact with experts in computational chemistry or drug delivery to significantly contribute to the design and development of innovative therapeutic approaches.

## 2. Computational Design of Anticancer Small Organic Molecules

Historically, the computational design of small organic molecules with pharmacological activity was carried out by two different approaches the ligand-based drug design (LBDD) and the structure-based drug design (SBDD). While LBDD is usually pursued when a significant number of molecules able to bind a given target is known [20], SBDD approach requires the knowledge of the three dimensional structure of the target. In the majority of the cases, the target structure is obtained by experimental techniques such as x-ray, NMR or Cryo-EM [21], however, the homology modeling technique can be used when the target structure is missing, but structures with sufficient level of homology (>25%–30%) are available [22].

To note, LBDD and SBDD are not rigidly separated and their integration contributed to the success of some studies where large libraries of compounds were screened [23].

The computational drug design process frequently requires the use of multiple techniques which accurate description is beyond the scope of this review. Therefore, we choose to suggest some good reviews and perspective to highlight the technical issues of the different techniques and to focus the attention on the general computational drug design process and on some selected applications.

Concerning the SBDD the applied computational techniques can be classified into three categories docking, MD simulations and free energy calculations.

Molecular docking algorithms are designed to predict the pose (i.e., the reciprocal orientation) and the affinity of a molecule towards a target of interest. Over the years, the possibility to include computationally more expensive approach and the improvement of the scoring function led to generally good performances in the prediction of the complex structure. However, in many cases, the prediction of the affinity for the target is still challenging and inaccurate. Multiple factors influence the quality of the structural predictions made by a docking software. Interestingly, the problematic inclusion of the reciprocal ligand-target adaptation during the binding process (‘induced-fit’ effect) and the possible role of structural water molecules in the formation of the complex seem to be more important than others [24].

Regarding the induced-fit effect, the first proposed technical solutions were based on the addition, in the docking protocol, of some steps devoted to the sampling of the possible conformation of selected side chains [25]. However, this type of approach does not allow to take into account larger structural rearrangements that involves the backbone structure, and thus, they cannot be suitable to study systems where the ligand binding induce large structural rearrangements.

Considering this limitation and taking advantage of the recent advancement in computer hardware and of the availability of new codes able to exploit the computer power offered by the graphic processor unit (GPUs), this problem is increasingly addressed by using a combination of docking and MD simulations [26] or with enhanced sampling simulations techniques such as Umbrella Sampling [27], steered MD [28], metadynamics [29] and supervised MD [30]. Running MD simulations for a sufficiently long time or coupling MD simulations with enhanced sampling techniques, enable the observation of rare events as ligand binding/unbinding, loop or channel opening/closure, protein folding.

In addition, the presence of crucial water molecules have been investigated by specialized algorithms as those implemented in the GOLD docking software [31] that permit to turn on/off the presence of a single water molecule inside the catalytic site or by the use of MD simulations [24,32,33,34].

Concerning the scoring of the ligand-protein affinity, many efforts have been devoted to improve the performance of the scoring functions, e.g., taking into account the ligand-target polarization effect by quantum mechanics/molecular mechanics QM/MM calculations [35,36], or using artificial intelligence to improve their general predictivity [10].

A significant contribution to the affinity predictions comes from the endpoint free energy methods such as MM-GB(PB)SA and Linear Interaction Energy (LIE). In both approaches, multiple conformations of the final state of systems are sampled by MD simulations, and the binding free energy is estimated by approximated expressions [12,37].

In particular, for MM-GB(PB)SA the difference in free energy (ΔG) is expressed as:

ΔG = Gcomplex − (Greceptor − Gligand)(1)

ΔG = ΔH − TΔS(2)

The enthalpic contribution ΔH can be decomposed in two contributions ΔG_MM_ and ΔG_solv._, ΔG_MM_ can be additionally decomposed in three contributions ΔG_vdW,_ ΔG_elc_ and ΔG_int_ that express the change in the van der Waals, electrostatic and e conformational energy, calculated at a molecular mechanics level. ΔG_solv_ can be expressed as the sum of ΔG_solv-pol,,_ which is the electrostatic contribution to the solvation energy, calculated applying the Poisson–Boltzmann or the Generalized Born equations, and ΔG_solv–nopol_ that it is estimated considering the variation the surface accessible area (SASA).

Since it is difficult to accurately estimate ΔS, it is frequently neglected when similar ligands are compared [38], however, it can be estimated by normal-mode analysis when the determination of a more rigorous ΔG is required.

Differently, in the LIE approach ΔG is expressed by Equation (3):

(3) $$\Delta G=\alpha 〈V_{Lprot}^{vdw}-V_{Lsolv}^{vdw}〉+\beta 〈V_{Lprot}^{elec}-V_{Lsolv}^{elec}〉$$

where $V_{Lprot}^{vdw}$ and $V_{Lsolv}^{vdw}$ are the average van der Walls and the electrostatic interaction energy of the ligand inside the protein or in the solvent and ⟨ ⟩ indicates that the quantity is calculated over a conformational ensemble (ensemble average). α, β represent empirical coefficients that depend on the nature of the system [39,40,41].

However, from the theoretical point of view, accurate results can be obtained only by the application of rigorous physical techniques such as Thermodynamic Integration (TI) or Free Energy Perturbation (FEP). For different reasons, such as the high computational costs and difficulties in obtaining convergent results for structurally unrelated compounds, these methods are still frequently applied only to the subtle optimization of compounds and not to the screening of small or large libraries. However, as recently pointed out by Williams-Noonan et al. [13], they are close to becoming a mainstream tool for medicinal chemists in the next few years.

### 2.1. Selected Examples of Anticancer Small Molecules Design

Scientific literature reports hundreds of studies where computational methods support the development of anticancer drugs [42,43]. Therefore, herein, we discuss only a few selected examples, one also from our research experience that can give the idea of how computational methods can be used in anticancer drug design.

One interesting example concerns the design of new human aromatase (HA) inhibitors. HA is a P450 cytochrome (CYP450) in charge for the conversion of androgens to estrogens and one of the main targets of the therapies against ER-positive breast cancer. Being that HA is a CYP450, it is characterized by a hidden catalytic site. Therefore in 2012, Sgrignani and Magistrato started to investigate the channels traveled by the substrate to enter/exit to/from the active site by computational methods [27,44,45]. In particular, after the generation of the first atomistic model of HA placed on a mimic of the endoplasmic reticulum membrane formed by 1-palmitoyl-2-oleoyl-sn-glycero-3-phosphocholine (POPC) molecules, they used random expulsion MD simulations (RAMD) to map an ensemble of putative channels. In fact, during RAMD simulations, a force of random direction and known intensity is imposed to the ligand and if this is able to move above a given distance threshold, in a given time interval, the direction is conserved, otherwise it is changed. As result of this procedure multiple unbinding events, in this case one hundred, can be sampled in a reduced simulation time. Finally, the unbinding trajectories have been clustered to identify some representative of really different enter/exit pathways and the steered MD (SMD) technique has been used to determine the most favorable. Differently than in RAMD, during SMD simulations, a force of know direction is imposed on the ligand in order to induce its distancing from the binding site at a constant velocity. This procedure allowed to calculate the work necessary to pull-out the ligand that has been used as measure of the accessibility of the channel. This work indicated that (1) the membranes’ environment significantly influence the results and it has to be considered in the modeling of HA and (2) two favorable access/release channels can be identified.

In 2017, thanks to the rapid availability of higher computational resources, Magistrato et al. [27] reconsidered their previous results and used umbrella sampling (US) simulations to obtain the free energy profile along the previously identified channels. This study indicated one of the channels as the most probable and contributed to the identification of structural rearrangement necessary for the passage of substrates and inhibitors.

Historically, HA inhibitors have been always designed as competitive ligands for the catalytic site and other never explored routes [46,47]. However, in 2014, inspired by biochemical studies carried out by the group of D. Flockhart [48,49,50] reporting the non-competitive inhibition of HA by some tamoxifen metabolites, Sgrignani et al. [51] also performed computational studies aimed to locate an allosteric site on the HA surface and to understand the mechanism of the non-competitive inhibition. The study started from the identification of some putative allosteric sites present on the HA surface made by the Sitemap software [52,53], then docking, MD and MM-GBSA simulations have been used to identify which sites were suitable to bind the tamoxifen metabolites with a predicted affinity consistent with the experimental data and to propose some mechanism of allosteric inhibition.

The information obtained from these studies, in particular those concerning the localization of the channels, have been later used to identify new allosteric or dual-mode (allosteric and orthosteric) HA inhibitors [54,55]. Specifically, they used docking based virtual screening, molecular dynamics and free energy calculations to identify some hits that represent the starting point for subsequent optimization studies. To note, the ability of the identified compounds to inhibit HA was proved by biochemical and cellular experiments [54,55].

Another interesting example about computer-aided anticancer design is represented by the study by Cherkasov and coworkers concerning the identification by virtual screening of one molecule able to inhibit the transcriptional activity of the ERG protein, a transcription factor identified to be highly expressed by prostate cancer cells. In this case, starting from the structure of the ETS-ERG, an ERG domain that has been proved to interact with DNA [56] directly, they localized a putative drug binding site using the Site Finder algorithm implemented in the Molecular Operating Environment (MOE).

Therefore, they processed a library of 20 million compounds obtained from ZINC (a large database of commercially available molecules) to select a sub-library of 3 million compounds with the following properties: molecular weight between 250 and 400 Da, logP ≤ 5, hydrogen-bond donors ≤5, hydrogen-bond acceptors ≤10, number of rotatable bonds ≤10 and number of rings ≤4. The ability of these compounds to bind ERG was evaluated by virtual screening using the Glide algorithm [57] and, only for top 30,000 molecules from this first calculations also by eHiTs docking program [58]. Finally, the poses of the best 3000 compounds (ranked by a consensus score) were visually analyzed and 48 molecules were selected for biological tests.

The obtained results indicated a molecule (VPC-18005) as the more promising compound. The interaction of this molecule with the target was finally proved by NMR spectroscopy and its effects on the prostate cancer cell investigated by molecular biology.

### 2.2. Computational Design of Anticancer Peptides

Peptides have been considered, for a long time, a niche area with reduced perspectives of development. This was mainly due to some intrinsic characteristic of this class of molecules such as inability to permeate cellular membranes, biological instability, low oral bioavailability and the capital roles played by peptides in the endocrine signaling [59]. However, it is widely recognized that they also offer multiple advantages, being well suitable to replace endogenous agonists or to target protein-protein interactions. Moreover, multiple strategies, such as the inclusion of unnatural amino acids, backbone modifications and novel formulations, allowed to overcome the previously discussed limitations giving a significant impulse to the peptide drug development.

Computational peptide design (CPD) is preferentially performed using a structure-based approach. The structures of protein-protein complexes are the major source of peptide sequences to be used in therapeutic peptide design, however, this type of information is not always available and then a great contribution can come from computational chemistry. Considering to have one or more bioactive peptides coming from random libraries, obtained from phage display or isolated from natural sources, for which not even a crude structural model is available, the first step to undertake a computational study aimed to improve the peptide affinity and selectivity for a given target is the building of a reliable model of the peptide-target complex. As a result of the larger conformational space explored by peptides with respect to small molecules, it is common opinion that the consolidated docking algorithms, widely used in drug design, are not suitable for this type of investigation, therefore specialized docking algorithms integrated, using experimental restrains, when it is possible, can help to identify the correct structure [30,60].

Once a structural model of the complex is available both MD simulations and/or free energy calculations demonstrated that they can significantly contribute to design peptide modifications that results in an improved affinity and selectivity [61,62]. In particular, starting from the assumption that a significant increment of the affinity for the target would result also in an augmented selectivity, multiple methodologies with different degree of complexity such as thermodynamic integration or free energy perturbation [63,64] metadynamics [65,66], MM-GB(PB)SA [5,67,68] aimed to estimate the binding free energy (ΔG) computationally, have been applied to evaluate which peptide modifications could result in a more favorable (more negative) binding energy. Additionally, in this case, literature reports some interesting examples about the use of computer simulations in the design of anticancer peptides, therefore we selected some of them to give to the reader a flavor of the impact that computer simulation had on this research field in the last ten years. Nevertheless, computer simulations of biological systems root in the 1970s [69], only the great technological progress such as computing by graphic processor units (GPUs) or the development of highly parallel codes [70,71] enabled computer simulations to really impact the peptide therapeutic development and to enrich the scientific literature in the last ten years.

### 2.3. Selected Examples of Anticancer Peptide Design

The work of Spodzieja et al. [72] represents an interesting example about the use of the structure of protein-protein complexes in computational design of anticancer peptides. In fact, their study was focused on the design of a peptide inhibitor of the herpes virus entry mediator (HVEM) protein, which is frequently expressed in melanoma cells and indicated as a possible target for anticancer immunotherapy. The visual observation of the structure of the complex’s structure of the complex between HVEM and the B and T lymphocyte attenuator (BTLA) protein suggested that a 17 residue aminoacidic extracted from HVEM (positions 23–39) could antagonize the formation of the HVEM-BTLA complex. This hypothesis was preliminarily verified by 10 ns long MD simulations that confirmed the ability of the peptide to bind BTLA. Experimental tests indicated that the peptide can really hinder the protein-protein interactions, however they also pointed out that this effect is mainly linked to the presence of a free cysteine in the peptide suggesting that the observed effect could be due to the formation of a covalent BTLA-peptide complex and not to the formation of a complex with the same structure observed in the X-ray experiments.

Another interesting example about the use of computational chemistry techniques to increase the affinity of peptides to a given target concerned the optimization of a 12-residue peptide modulator (L-peptide, sequence: RLLDTNRPLLPY). This peptide was firstly identified by phage display experiments [73], carried out to identify peptides able to specifically bind Nasopharyngeal carcinoma (NPC) cells. Nevertheless, even if this peptide was designed to bind specific cells, showing a clear biological effect, its molecular target was unknown. Therefore, some years after its first publication, Yu and coworkers applied the PepBind program [74] to identify the molecular target of the peptide, concluding that the human glucose-regulated protein 78 (GRP78) protein could be the molecular counterpart of the L-peptide. GRP78 is a protein located in the endoplasmic reticulum (ER), where it is involved in the promotion of protein folding and in the activation of unfolded protein response pathway (UPR). Due to high ER stress level, this protein is highly expressed on the surface of many cancer cell lines and, therefore, it is a promising target for the development of innovative anticancer therapies. At the beginning of the study, the structure of GRP78 was still unknown; therefore computational studies, aimed to optimize the peptide, started with the generation of the structural model of the target by homology modeling considering different proteins such as DnaK and Hsc70 as the template [75]. Subsequently, the Dock [76] program and the Hotlig suite [77] were, respectively, used to produce a structural model of the protein-peptide complex and to analyze the interactions between the two molecules. The structural model of the protein-peptide complex was finally used to design a library of 400 peptides to be screened in-silico for their ability to bind GRP78. The performed calculations identified a group of 17 peptides that could bind the target better than the original L-peptide. These molecules were synthetized and their ability to bind GRP78 verified by surface plasmon resonance (SPR) experiments. Four peptides, approximately the 25% of the tested molecules, displayed a better affinity for the target.

**Figure 1:**
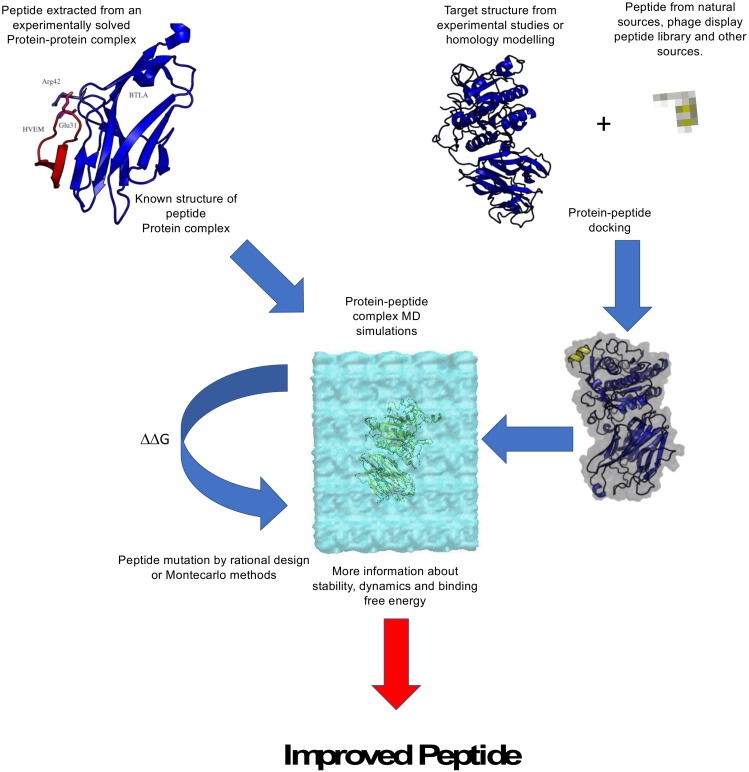
Schematic representation of a computational pipeline for peptide optimization.

In recent years, some of the authors of this review applied the combination of the structural modeling and MM-GBSA calculations to the optimization of peptides able to inhibit the Proprotein convertase subtilisin/kexin type 9 (PCSK9). The evidence about the relevance of PCSK9 inhibition to develop anticancer therapies is still not clear, while it is well known that this protein is one of the main targets for the therapies against hypercholesterolemia [78]. However, the strategy pursued to optimize one peptide (T9) selected by the screening of peptide molecules derived from the hydrolysis of lupin protein was successful and therefore could represent a suitable strategy also to optimize the affinity of anticancer peptides (Figure 1). PCSK9 regulates the low-density lipoprotein receptor (LDLR) degradation by direct interaction with this protein. In particular, it has been demonstrated that gain of functions (GOF) mutations able to increase the PCSK9-LDLR affinity result in an augmented LDLR degradation that has as final effect a significant increment of the circulating cholesterol concentration.

The T9 peptide (GQEQSHQDEGVIVR) was firstly identified, together with other peptides, in a study aimed to investigate the ability of a group of molecules obtained from the hydrolysis of lupin protein to suppress the PCSK9-LDLR interaction [5]. Further studies pointed out that, differently from the other peptides reported in the original study, T9 is able to suppress also the binding between the mutated PCSK9^D374Y^ and the LDLR even with an EC50 value of 285.6 ± 2.46 μM [79].

In all these studies computational techniques have been used to obtain a structural model of the PCSK9-LDLR complex; moreover, in the case of T9, a specific computational protocol was applied also to improve it affinity for PCSK9^D374Y^ [62].

In particular, computational alanine scanning calculations, based on the MM-GBSA technique [80,81] were firstly used to identify the “non-hot-spot” residues, e.g., the residues not essential for the binding. Interestingly, these calculations led to the discovery that the simple substitution of the aspartate residue in position 8 of T9 with an alanine could result in an improved affinity. This prediction was confirmed by in-vitro experiments.

MM-GBSA calculations identified also other additional non-hot-spot residues, therefore starting from the T9D8A structure 10.000 new peptides were generated by changing the residues at the identified positions with all the other natural amino acids but accepting only two simultaneous mutations. The affinity for the target of these new residues was firstly evaluated by the scoring function implemented in Prime (Prime, Schrödinger, LLC, New York, NY, USA), therefore the structures of the complexes between PCSK9^D374Y^ and the top-ranked peptides were simulated by MD for 100 ns and the peptide-protein affinity estimated by MM-GBSA. However, the results of these calculations indicated that none of the hypothesized mutations could actually improve the affinity for the T9 binding when compared to other positions more crucial for the T9 binding and not initially classified as non-hot-spot residues, when investigated using the same computational protocol. Finally, a new T9 derivative (T9D8A_1) was identified and the in-vitro test confirmed that the EC_50_ able to block the T9 - PCSK9^D374Y^ is reduced by more than the 50% compared to the original T9 peptide.

## 3. Advanced Drug Delivery Approaches

In the following chapter, we will discuss a range of drug delivery strategies, and focus on some from our research experience, given their potential for targeted therapies.

### 3.1. Oncolytic Viruses in Drug Delivery

**Figure 2:**
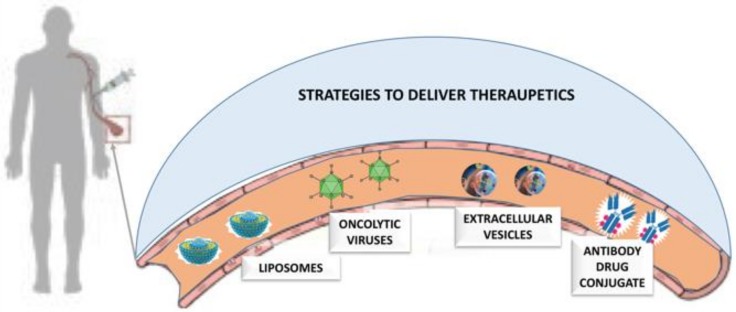
Innovative drug delivery strategies.

Cancer continues to be one of the most difficult global healthcare problems. Although there is a large group of drugs that can be used in cancer treatment, the problem is the selective killing of cancer cells while reducing collateral toxicity to healthy cells. In addition to incrementing the affinity to the macromolecular target, drug selectivity can also be incremented by innovative drug delivery strategies (Figure 2).

Concerning the oncological therapies, one of the most important sources of resistance to chemotherapy, apart the ability to develop cellular mechanism of drug resistance, is the tendency of some tumors to proliferate in the so-called sanctuary sites (i.e., central nervous system, peritoneum and testis) [82], anatomical spaces where the drugs cannot reach the therapeutic levels. Moreover, an indiscriminate distribution of chemotherapeutic drugs also to healthy tissues is cause of side effects. To overcome these issues, various drug delivery systems have been extensively investigated demonstrating that they can effectively improve the selectivity and safety of the drugs [83]. However, the precise delivery of active drugs to cancer tissues is still challenging [84] and this issue still represents an unmet medical need.

In the following, we will discuss some very interesting drug delivery strategies such as oncolytic viruses (OVs), liposomes, antibody drug conjugate and extracellular vesicles.

OVs are an emerging class of anticancer bio-therapeutics able to selectively auto-replicate in cancer cells without causing damage to normal cells [85,86]. They can be either naturally tumor-selective viruses, like reovirus and Seneca Valley virus, or genetically modified so that their replication is dependent on cancer-related pathways, such as adeno, vaccinia, herpes simplex, measles and Newcastle disease viruses. Furthermore, OVs have shown a potential for the treatment of cancer along with the safety profile as verified in many preclinical studies and clinical trials [87,88,89].

Importantly, the US Food and Drug Administration (FDA) and European Medicines Agency (EMA) in 2015 have approved T-VEC (Imlygic), an oncolytic herpes virus lacking ribonucleotide reductase and also expressing granulocyte-macrophage colony-stimulating factor (GM-CSF), for the treatment of locally advanced or non-resectable melanoma, opening the way for new therapeutic protocols [90]. However, clinical data highlight that the efficacy of OVs as single agent remains limited, suggesting that patients may benefit from combination therapies. In fact, OVs exhibit different mechanisms of action from conventional anticancer approaches giving a possibility for additive or synergistic interactions in cancer therapy [91,92,93]. Nevertheless, there has been huge progress with anti-cancer therapies with encouraging clinical results, there are still many limitations that should be tackled to improve the efficacy of oncolytic virotherapy [94,95,96] including: viral tropism, delivery platforms, viral distribution and oncolysis by the OVs. Although the greatest effect of OVs consists of their selective infection and replication in cancer cells, the ability to deliver OV particles efficiently to tumors still constitutes a hurdle. In fact, in order to establish a niche within the tumor after a systemic injection, OVs have to bypass the liver that could sequester a percentage of the administered dose. Therefore, administering the virus directly within the tumor overcomes this limitation, reducing the possibility of treating solid tumors where the logistics of intralesional administration may prohibit its use. In addition, as most OVs are ubiquitously present in nature and humans could have been infected or vaccinated against some, patients might have neutralizing antibodies that can bind the virus, reducing the viral spread and limiting the targeted delivery [97]. In addition to that, the OV-efficacy in monotherapy is limited, therefore, in order to enhance their efficacy, novel approaches able to combine OVs with anticancer agents are remarkably increasing [98]. In addition to the possibility of a combination regime in which OVs are administered with free drugs, another approach has been proposed to absorb anticancer drugs, for example, L-carnosine, on the adenoviral capsid, based on electrostatic interaction and exploited this strategy for cancer drug delivery [99,100]. The studies were designed to evaluate the oncolytic potency of OVs and its anti-cancer efficacy in combination with L-carnosine in human colorectal and lung cancer animal models revealing that the intratumoral administration of the complex (OVs complexed with L-carnosine) resulted in significant synergistic tumor suppression [100].

Considering that OVs have a unique ability to selectively replicate in cancer cells, a clinically relevant strategy, able to accelerate their cytotoxic properties and improve oncolysis, is their combination with chemotherapeutics [101]. Therefore, since chemotherapy can enhance the replication of oncolytic viruses and weaken the immunosuppressive tumor microenvironment [102], a clinically highly relevant strategy is the use of OVs with chemotherapeutic agents which can enhance anticancer killing efficacy and induce anticancer immunity [91]. For example, combining the oncolytic virus ONCOS-102 with chemotherapy [91,93,103] can overcome the immune suppressive tumor microenvironment [104] due to the immunogenic tumor cell death induction properties [105,106,107,108] and subsequently mediation of anti-cancer immune responses [105,109,110]. In fact, emerging findings suggest that the clinical outcome of conventional chemotherapy is not only attributed to tumor cell toxicity but also results from the induction of anti-cancer immune responses. Antitumor immune response can be primed by immunogenic cell death (ICD), which is characterized by cell-surface translocation of calreticulin (CRT), extracellular release of ATP, high mobility group box 1 (HMGB1) and stimulation of type I interferon (IFN) responses. The conventional chemotherapeutics can work as ICD inducers, which are capable of modulating tumor-infiltrating lymphocytes (TILs) and reactivating antitumor immunity within an immunosuppressive microenvironment. Such immunological effects of conventional chemotherapy are critical for the better prognosis of cancer patients [111].

OVs can serve as an immunesensitizer in combination therapies with checkpoint inhibitors [112]. The discovery of immune checkpoint inhibitors (ICIs) such as anti- CTLA-4, PD-1 and PD-L1 is a revolutionary milestone in the field of immuno-oncology, holding promising therapeutic outcomes [113]. Tumor cells evade immunosurveillance and progress through different mechanisms, including the activation of immune checkpoint pathways that suppress antitumor immune responses. Checkpoint inhibitors have revolutionized the treatment of advanced melanoma by releasing suppression of T-cell immunity in the tumor milieu, by inducing durable clinical antitumor immune response and supporting long-term survival in patients [114]. Nevertheless, despite the success of ICI therapies, only a fraction of patients benefit from it with the best response rates that do not exceed 35% to 40% [115,116]. However ICIs have the capacity to synergize with other approaches to activate anti-cancer immune responses [117]. For example, a study in humanized mice engrafted model with A2058 melanoma cells showed significant tumor volume reduction after ONCOS-102 treatment. Importantly, the combination of pembrolizumab with ONCOS-102 reduced tumor volume to an even greater extent, while pembrolizumab alone did not show any therapeutic benefit by itself supporting the scientific rationale for the combination therapies [92].

Future studies on oncolytic virotherapy should focus on innovative strategies and overcome current challenges and limitations by using recombinant OVs to create solid foundations for future clinical success.

### 3.2. Liposomes

Liposomes are phospholipid-based vesicles, with a diameter range of 50–200 nm for parenteral administrations, in which the circular bilayer creates an aqueous core. This structure offers the possibility to entrap both hydrophilic molecules, hosted in the aqueous space, and hydrophobic molecules, inserted within the lipid bilayer. They possess unique properties owing to the amphiphilic character of the lipids and several advantages such as biodegradability and biocompatibility which makes them suitable delivery systems [118], furthermore, their composition, surface charge and functionalization, size and other physicochemical features can be controlled in order to modulate biological processes such as drug release, circulation time and biodistribution [119,120]. Liposomes emerged as sustained drug release systems able to increase the delivery of therapeutic agents to the tumor site and reduce the off-target toxicity of the anticancer drug. Liposomes have been firstly investigated as drug carriers in 1973 [121], leading to considerable improvements in pharmacokinetics of the encapsulated drug, slowing the rate of drug clearance and displaying a minor tendency to localize in healthy tissues when compared to the free drug.

Liposomal drug delivery systems enable the delivery of higher drug concentrations allowing targeting specific cells or organs [122,123], thus minimizing the distribution of the drug to non-targeted tissues. Nevertheless, the uptake by the mononuclear phagocyte system, in liver and spleen, represents the principal mechanism of liposomal clearance from the bloodstream, thus preventing the full exploitation of the advantages of this drug delivery system. To circumvent this issue, the anchoring of hydrophilic polymers has been proposed, such as PEG able, to the surface of the liposomes and create a barrier against the adherence of opsonins that can enhance phagocytosis. Stealth^®^ liposomes, with PEG on the outer membrane, display steric stabilization and low opsonization with reduced uptake and clearance by the mononuclear phagocyte system. These liposomes have an extended circulation time and represent an efficient carrier able to reduce the mononuclear phagocyte system uptake while supporting the delivery of therapeutic drugs to cells or tissues [120,124].

Currently, several liposomal formulations have been used in clinic, including the first FDA-approved Doxil^®^, a PEGylated doxorubicin liposome formulation for the treatment of solid tumors, and Marqibo^®^, the latest FDA approved liposome formulation which is a sphingosomal/cholesterol encapsulation of vincristine sulphate for the treatment of Philadelphia chromosome-negative acute lymphoblastic leukemia [125].

In a recent work aimed to treat glioblastoma, PEGylated liposomes have been employed for the delivery of resveratrol in order to improve solubility and stability [126]. Using transferrin for the active targeting, transferrin receptors are upregulated in glioblastoma. In addition, rhodamine-labeled transferrin-modified liposomes demonstrated higher association with cancer cells compared to human astrocytes, with significant internalization in cancer cells compared to non-targeted liposomes. Interestingly, resveratrol-loaded transferrin-modified PEGylated liposomes inhibited tumor growth and improved survival in mice suggesting their application could be a valuable strategy for the treatment of glioblastoma.

So far, clinically approved liposome drug formulations do not include specific active-targeting strategies (Doxil^®^/CaelyxTM, DaunoXome^®^, Myocet^®^, Marqibo^®^, Onivyde^®^, VyxeosTM); however, a doxorubicin-loaded immunoliposome targeted with Cetuximab (mAb epidermal growth factor receptor (EGFR) inhibitor) Fab fragments is in Phase II clinical trials for the treatment of advanced triple-negative EGFR-positive breast cancer (Clinical trial NCT02833766). Future directions may include some strategies for the co-administration of two targeted formulations or the combination of two drugs within a single targeted carrier.

### 3.3. Extracellular Vesicles and Cancer Drug Delivery

In the last decades, extracellular vesicles (EVs) have been found to exert different biological functions in both physiological and pathological conditions explaining the rapid growth of interest in this research field. EVs are nano- to micron-sized lipid membrane-bound vesicles secreted into the extracellular environment transporting proteins, lipids and nucleic acids from cell to cell [127]. They are naturally occurring cargo delivery agents with the potential to be used as vehicles for drug delivery [128,129]. Due to their natural origin, their efficient internalization by recipient cells and their natural ability to target specific cells or organs [130,131], they are considered as ideal carriers for therapeutic agents. Hong et al. developed engineered enzymatic EVs with the native glycosylphosphatidylinositoll (GPI)-anchored form of hyaluronidase (Exo-PH20) to overcome the immunosuppressive tumor microenvironment [132] showing that the EV-mediated codelivery of PH20 hyaluronidase and a doxorubicin efficiently inhibits tumor growth.

Currently, different strategies can be used to load the therapeutic cargo within the EVs. The cargo can be loaded into EV producing cells or post-EV formation by electroporation, passive incubation or destabilizing the EV membrane [131,133]. A variety of therapeutic agents have been loaded into EVs in order to increase their accumulation inside target cells [134,135,136]. For example, curcumin and doxorubicin exhibited a high drug loading efficiency and an increased cytotoxic effect when loaded inside EVs compared with the free drugs [137,138]. Due to their natural origin, EVs have recently had considerable attention as non-immunogenic drug delivery vehicles [130,139], and the systemic (i.v. or i.p.) route of administration was chosen in different in vivo preclinical studies [140,141,142]. In fact, recent studies report that the systemic delivery of cancer derived EV-formulations containing OVs were effective in reducing lung tumor growth in nude mice and were able to protect the virus from immune disruption through the encapsulation into EVs, suggesting that the procedure could protect the OVs against their disruption by the host immune system, while efficiently targeting the therapeutic particles into the neoplastic tissue [135].

The organotropism of EVs, the absence of immunogenicity, natural composition and ability to be loaded with small molecules and biologics are of interest for the development of nano-based drug delivery systems (DDSs) and theranostics applications [125,143,144]. Nevertheless, although highly attractive, the use of EVs in clinical applications remains limited because the molecular basis of their tropism and targeted delivery to cancer cells has not been thoroughly elucidated. Several studies reported that the tumor tropism of EVs might be related to the adhesion-associated molecules on their surface, such as integrins, tetraspanins and other glycoproteins [145]. However, there is no clear consensus whether the tumor tissue originating the EVs dictates their tropism [146]. Furthermore, bioluminescence and fluorescence imaging technologies indicate a selective delivery of EVs to the tumor tissue allowing to insulate the delivered agents, thus potentially preventing undesired off-target effects of the transported drugs due to their systemic delivery [147]. Interestingly, another advantage of using EVs is their heterologous and cross-species cancer specific homing capabilities that could open new avenues for the selective delivery of diagnostic/therapeutic agents to different tumor types [127]. The possible existence of a selective ligand-receptor mechanism responsible for the EV-tumor tropism paves the way for future research aimed at developing biocompatible nanovesicles with a cancer-selective homing which is instrumental for future clinical applications where the delivery of diagnostic agents may be combined with the loading of drugs or radiotherapeutic isotopes for curing tumor micrometastasis and residual neoplastic cells eventually remaining in the normal tissue after conventional cancer treatments [148].

### 3.4. Antibody-Drug Conjugates

Antibody drug conjugates (ADCs) are a class of anticancer therapeutics based on the direct conjugation of drug molecules to proteins in order to target the delivery of drugs to precise site of action [149]. The first ADC’s study linked the anticancer drug methotrexate to a polyclonal leukemia cell-targeting antibody in an attempt to target diseased tissue while leaving healthy tissue unharmed [150].

The first clinical trial with an ADC, conducted in 1983 by Ford and colleagues, employed an anti- carcinoembryonic antigen-antibody conjugated to vindesine, which is a vinca alkaloid, for the treatment of patients with advanced metastatic colorectal or ovarian carcinoma [151] revealing, by detecting radioactivity from the 131I labeled ADC, that five of the eight treated patients showed a clear localization of the ADC at the tumor site. An important translational aspect is related to the amount of ADC that cell receives. Clinical observations for many ADCs, such as ado-trastuzumab emtansine and mirvetuxi-mab soravtansine in breast and ovarian cancer patients, support the concept that the internalization by the cell, with accumulation of the cytotoxic activity, is directly related to target antigen density. Therefore, ADC therapy may be optimized by selecting patients whose tumors express target antigens above a threshold level necessary for antitumor activity [152]. To date, four ADC therapeutics received marketing approval: i) Mylotarg [153], gemtuzumab ozogamicin targeting the CD33 antigen was approved in 2000 for treatment of acute myeloid leukemia, and later on withdrawn in 2010 by the FDA as it failed to meet the efficacy targets; Mylotarg was re-introduced in 2017 with lower recommended dosage and different treatment schedule (alone or in combination with chemotherapy) for newly-diagnosed CD33-positive acute myeloid leukemia patients; ii) Adcetris – Brentuximab [154], vedotin targeting the CD30 antigen has been approved for the treatment of relapsed or refractory Hodgkin lymphoma and systemic anaplastic large cell lymphoma and several phase III trials are in progress to confirm clinical benefit of Brentuximab in randomized studies in combination with approved chemotherapeutic agents (NCT01712490, NCT01777152), as well as in combination with immune checkpoint inhibitors (NCT02684292, NCT03138499); iii) Kadcyla [155]-Trastuzumab-emtansine targeting the HER2/neu receptor for the treatment of HER2-positive metastatic breast cancer; iv) Besponsa-Inotuzumab [156] ozogamicin targeting the CD22 antigen used for the treatment of relapsed or refractory B-cell precursor acute lymphoblastic leukemia. The successful approvals of brentuximab vedotin, ado-trastuzumab emtansine and inotuzumab ozogamicin after decades of disappointment in developing immunoconjugates as therapeutic agents have attracted a lot of attention and more than 80 ADCs have entered clinical evaluation over the last 15 years [157].

However, the relatively benign side-effect profiles for many ADCs suggest that it could be combined with other agents in order to improve patient outcomes. In fact, mirvetuximab soravtansine is being evaluated in combination with carboplatin, pegylated liposomal doxorubicin, bevacizumab or pembrolizumab, in a trial called FORWARD II (NCT02606305) [158]. Furthermore, it has been suggested that the ADCs may be combined with immune checkpoint inhibitors such as the anti-PD1 antibodies (pembrolizumab, nivolumab) for enhanced and sustained antitumor effect [159].

Nevertheless, the large variety of ADC technologies developed over the past decade provides different possibilities for designing an ADC specific to a given target [157]. There are new-generation linker chemistries that result in improved antitumor activity and a wider therapeutic window in preclinical studies when compared with the approved ADCs [160,161,162]. In addition to that, there are still important parameters which need to be optimized such as the ADC stability which includes the rate of drug loss due to linker instability and the impact of conjugation on the antibody itself that can affect the pharmacokinetic and biodistribution. Interestingly, the instability of ADCs in the circulation may also be overcome by alternative bioconjugation chemistries with the aim to enhance homogeneity and to reduce the drug deconjugation rate in the circulation in order to limit off-target toxicity while increasing the delivery of drugs to tumors.

Future developments to increase the therapeutic index of ADCs have to be made either in the potency of the cytotoxic agent to reduce the minimum effective dose or improve the tumor selectivity while increasing the maximum tolerated dose. Currently, there is still a lot to learn about the optimal application of ADCs in the treatment of different cancers, especially in establishing the role of ADCs in combination with immune-oncology therapies. Nevertheless, the opportunity to improve treatment outcomes for cancer patients by incorporating ADCs into cancer therapy offers exciting possibilities for the future.

## 4. Computational Chemistry in the Design of DDS

For many years, delivery systems such as OVs, liposomes, antibody drug conjugate and extracellular vesicles have been considered to have too large dimensions for investigations using MD simulations. In fact, the simulation time, which is required to run MD simulations, directly depends on the number of atoms, that for such complex systems can frequently be also some millions.

Therefore, for some years, computational studies have been mainly focused on some relevant aspects for the drug delivery field such as the permeation of a drug across a membrane bilayer by using simplified systems [18,163,164,165] However, recent advances, as the computing by graphic processor units (GPU) and the availability of atomistic or coarse-grain force field, enables to investigate these systems accurately and in a reasonable time.

The development of antibody drug conjugates appears to be the most mature field for the application of computational methods [166] since docking, molecular dynamics and free energy calculation can be used to both speed-up the design of specific antibodies [167] and to investigate the interaction of the antibody with an additional carrier [168].

Concerning liposomes, Hashemzadeh et al. [169] recently reported the use of coarse-grain MD simulation to investigate the behavior of liposomes formed by 1,2-distearoyl-sn-glycero-3-phosphocholine (DSPC) and DPSM (Egg sphingomyelin). The results of the simulations indicated that even if DSPC and DPSM are usually used to generate liposomes and micelles, during the simulations of the self-assembly process, they form some nanodisc structures. This information can be useful in the future for further development of liposomal based formulations.

Regarding OVs, the computational hardware, currently available in the majority of the computational chemistry labs, cannot sufficiently simulate so large and complex systems, nevertheless some researchers demonstrated that the simulation of an entire viral capsid is possible [170,171] suggesting that, by improving the computational power, the computational design of very complex systems, such as OVs, will be routinely accessible.

## 5. Conclusions

Targeted cancer therapy relies on DDS able to deliver anticancer agents safely to tumors while minimizing systemic toxicity. This goal can be pursued by the identification of highly selecting compounds or developing strategies that maximize drug concentration in tumor compared to healthy tissue. The examples discussed in this review demonstrate that computational chemistry is becoming more and more important in improving the potency and selectivity of anticancer drugs and that advanced drug delivery techniques can significantly contribute to the development of new therapeutic approaches.

For many years the lack of computational resources limited the interaction between the two disciplines to which this review is dedicated, consequently computational strategies are not widely used for the design of DDS. In particular, most of the computational studies focused on specific aspects of the drug delivery development such as the calculation of the drug physicochemical properties or membrane permeability processes instead of studying the entire DDS.

Nevertheless, as science is evolving, we expect that in the next few years computer aided DDS design (CADDSD) should be increasingly combined with wet-lab techniques to facilitate the design of new therapeutic strategies aiming to (1) predict better and better the behavior of DDS under physiological environment, and (2) to design molecules able to drive the DDS at the right place to maximize targeting efficiency and minimize drug dosage.
